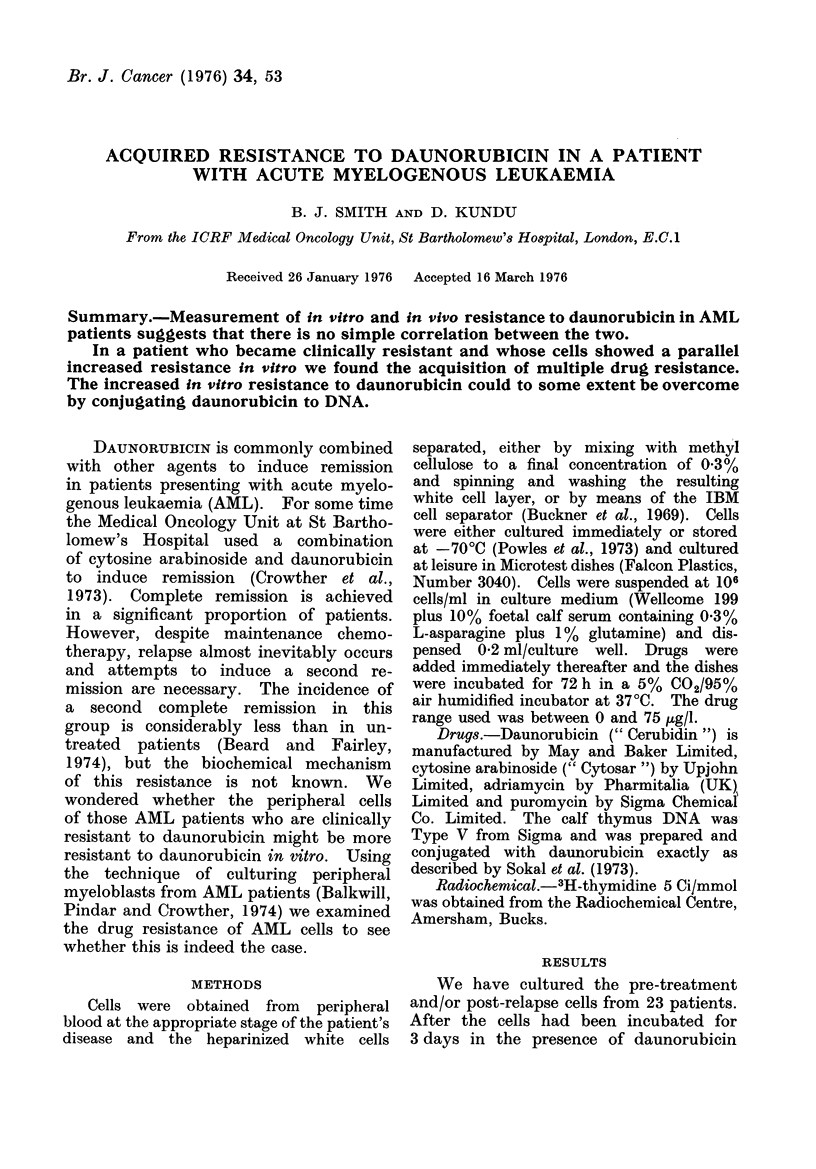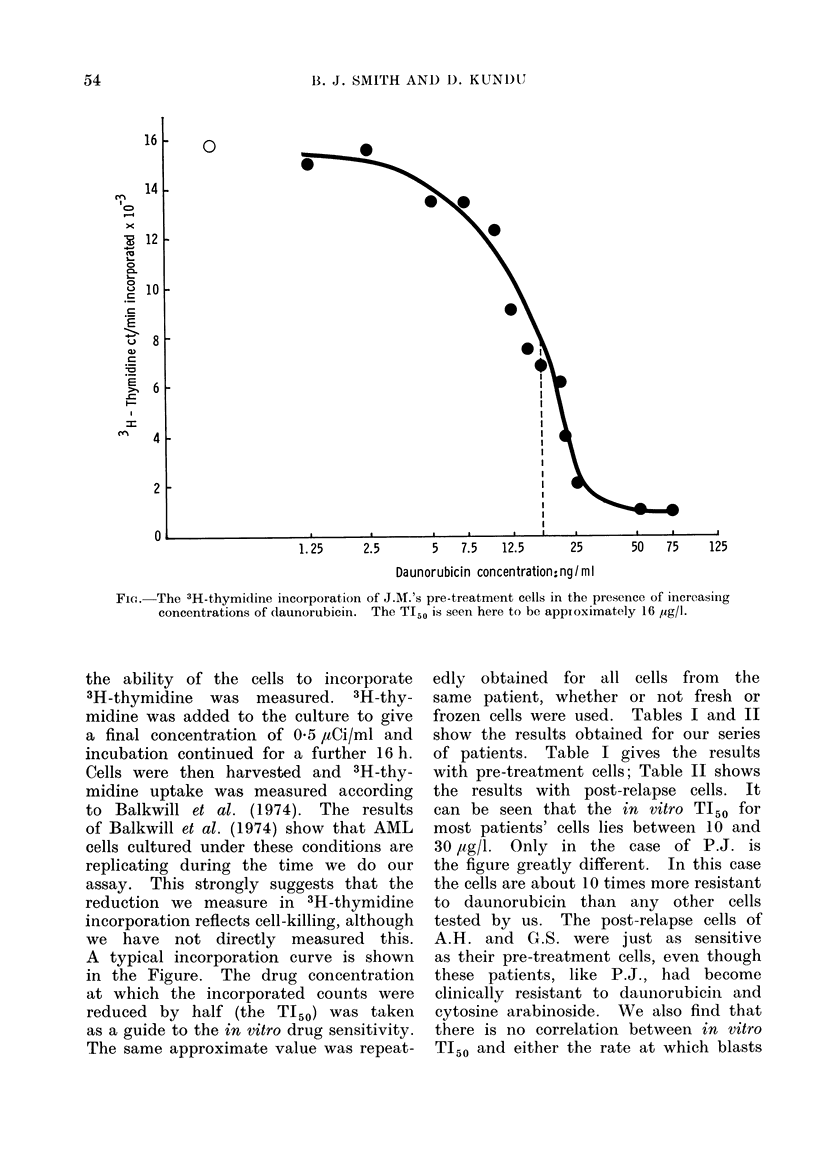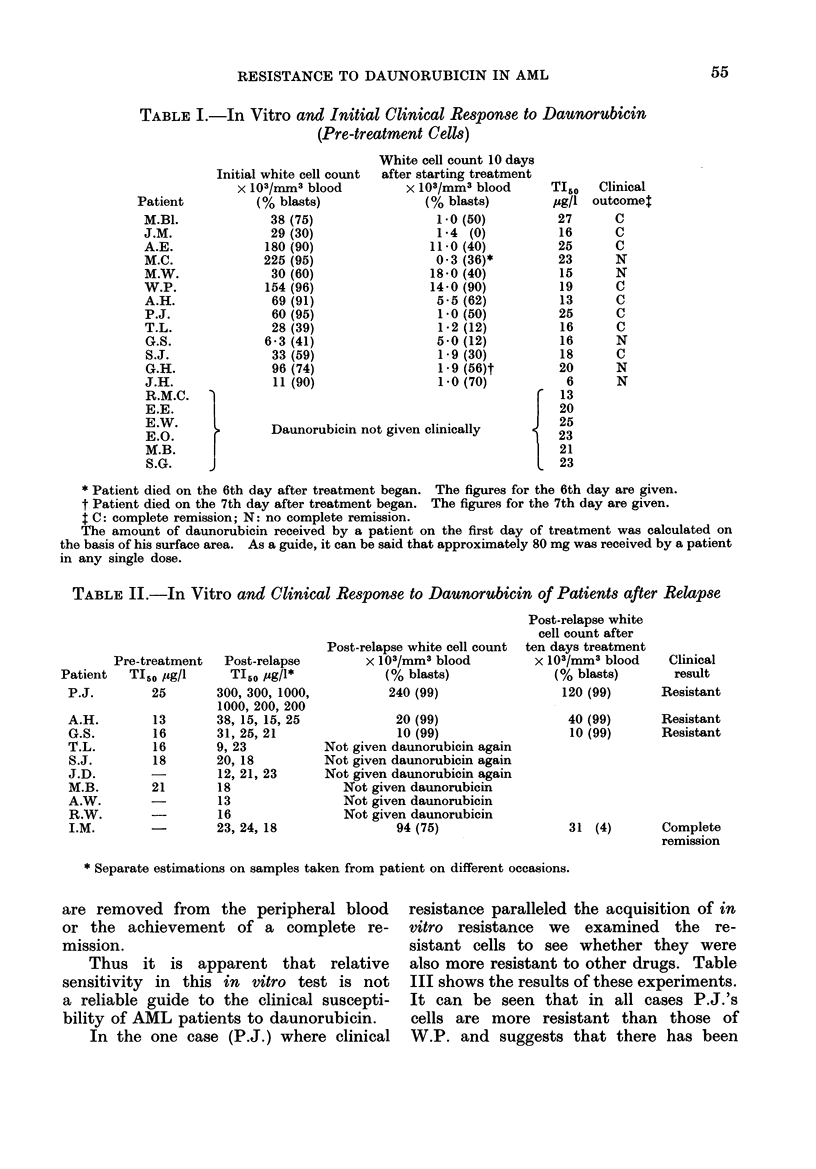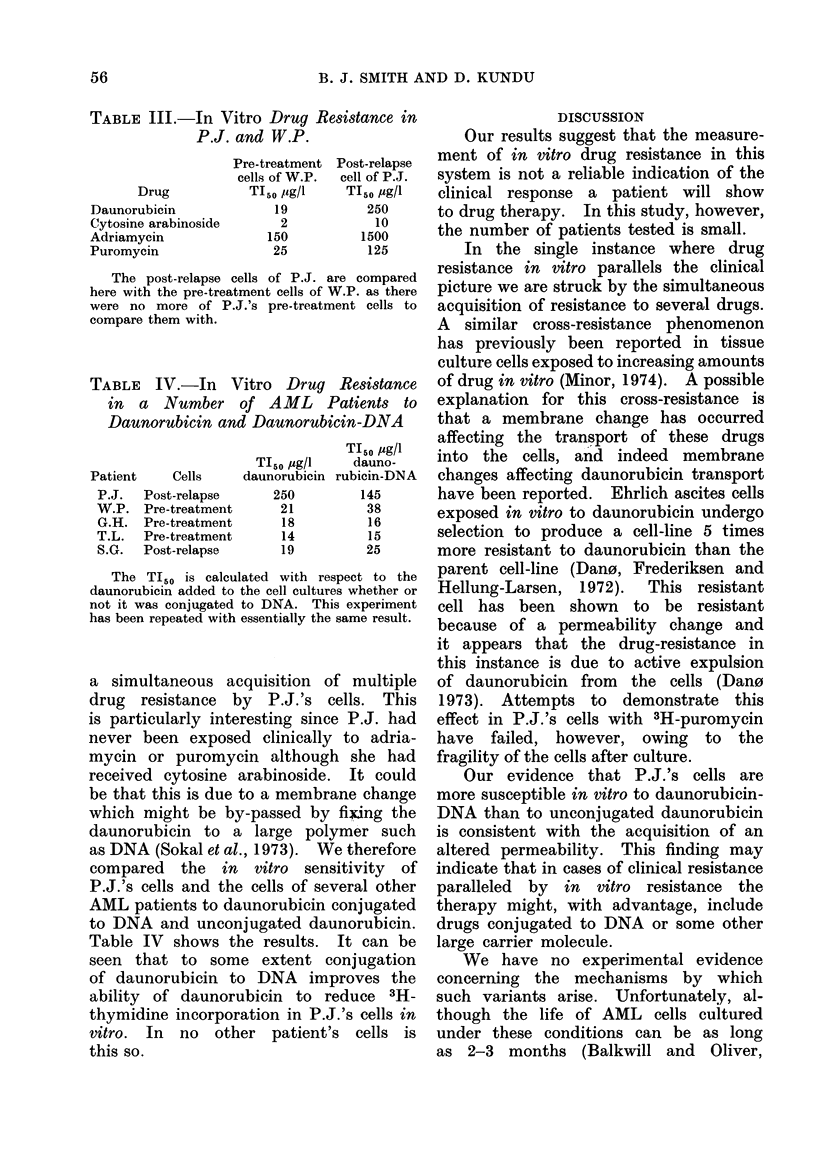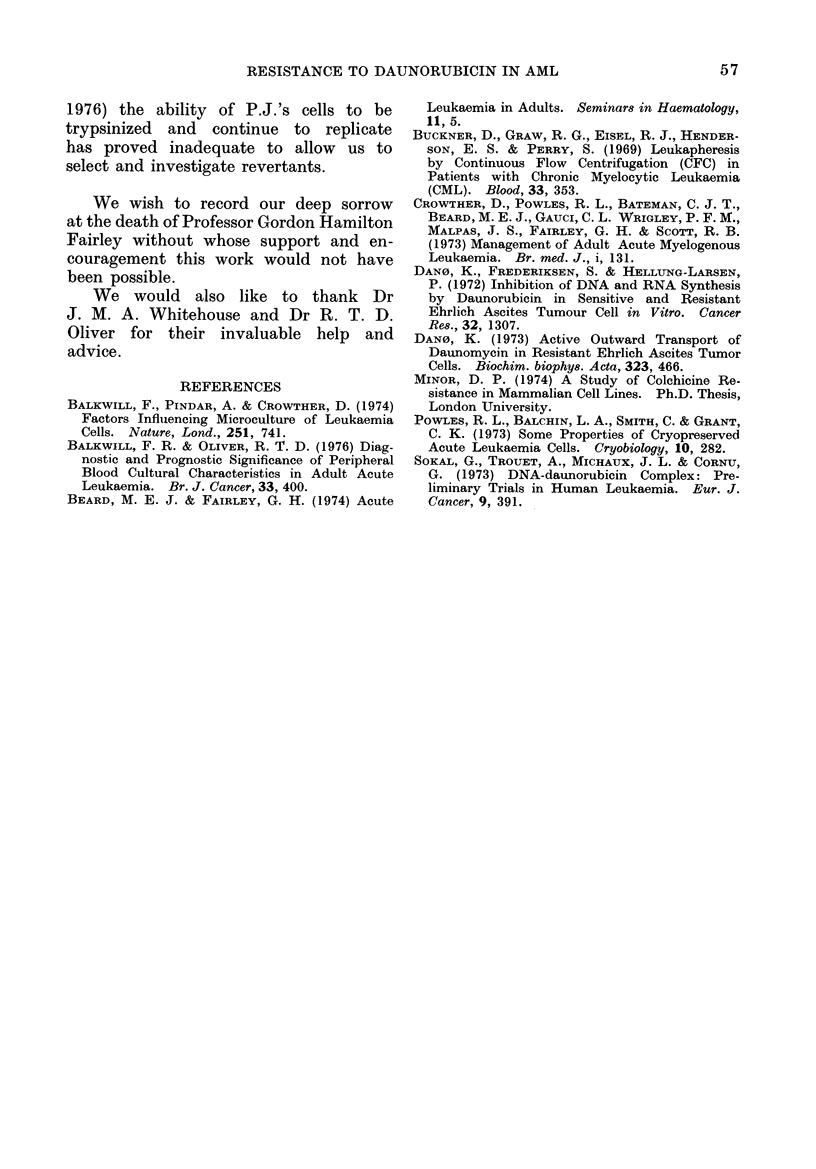# Acquired resistance to daunorubicin in a patient with acute myelogenous leukaemia.

**DOI:** 10.1038/bjc.1976.121

**Published:** 1976-07

**Authors:** B. J. Smith, D. Kundu

## Abstract

Measurement of in vitro and in vivo resistance to daunorubicin in AML patients suggests that there is no simple correlation between the two. In a patient who became clinically resistant and whose cells showed a parallel increased resistance in vitro we found the acquisition of multiple drug resistance. The increased in vitro resistance to daunorubicin could to some extent be overcome by conjugating daunorubicin to DNA.


Br. J. Cancer (1976) 34, 53

### ACQUIRED RESISTANCE TO DAUNORUBICIN IN A PATIENT WITH ACUTE MYELOGENOUS LEUKAEMIA

B. J. SMITH AND D. KUNDU

*From the ICRF Medical Oncology Unit, St Bartholomew's Hospital, London, E.C.1*

Received 26 January 1976 Accepted 16 March 1976

**Summary.—Measurement of *in vitro* and *in vivo* resistance to daunorubicin in AML patients suggests that there is no simple correlation between the two.**

**In a patient who became clinically resistant and whose cells showed a parallel increased resistance *in vitro* we found the acquisition of multiple drug resistance. The increased *in vitro* resistance to daunorubicin could to some extent be overcome by conjugating daunorubicin to DNA.**

DAUNORUBICIN is commonly combined with other agents to induce remission in patients presenting with acute myelogenous leukaemia (AML). For some time the Medical Oncology Unit at St Bartholomew's Hospital used a combination of cytosine arabinoside and daunorubicin to induce remission (Crowther *et al.*, 1973). Complete remission is achieved in a significant proportion of patients. However, despite maintenance chemotherapy, relapse almost inevitably occurs and attempts to induce a second remission are necessary. The incidence of a second complete remission in this group is considerably less than in untreated patients (Beard and Fairley, 1974), but the biochemical mechanism of this resistance is not known. We wondered whether the peripheral cells of those AML patients who are clinically resistant to daunorubicin might be more resistant to daunorubicin *in vitro*. Using the technique of culturing peripheral myeloblasts from AML patients (Balkwill, Pindar and Crowther, 1974) we examined the drug resistance of AML cells to see whether this is indeed the case.

### METHODS

Cells were obtained from peripheral blood at the appropriate stage of the patient's disease and the heparinized white cells separated, either by mixing with methyl cellulose to a final concentration of 0·3% and spinning and washing the resulting white cell layer, or by means of the IBM cell separator (Buckner *et al.*, 1969). Cells were either cultured immediately or stored at −70°C (Powles *et al.*, 1973) and cultured at leisure in Microtest dishes (Falcon Plastics, Number 3040). Cells were suspended at $10^6$ cells/ml in culture medium (Wellcome 199 plus 10% foetal calf serum containing 0·3% L-asparagine plus 1% glutamine) and dispensed 0·2 ml/culture well. Drugs were added immediately thereafter and the dishes were incubated for 72 h in a 5% $CO_2$/95% air humidified incubator at 37°C. The drug range used was between 0 and 75 μg/l.

*Drugs.*—Daunorubicin ("Cerubidin") is manufactured by May and Baker Limited, cytosine arabinoside ("Cytosar") by Upjohn Limited, adriamycin by Pharmitalia (UK) Limited and puromycin by Sigma Chemical Co. Limited. The calf thymus DNA was Type V from Sigma and was prepared and conjugated with daunorubicin exactly as described by Sokal *et al.* (1973).

*Radiochemical.*—$^3$H-thymidine 5 Ci/mmol was obtained from the Radiochemical Centre, Amersham, Bucks.

### RESULTS

We have cultured the pre-treatment and/or post-relapse cells from 23 patients. After the cells had been incubated for 3 days in the presence of daunorubicin

FIG.—The $^3$H-thymidine incorporation of J.M.'s pre-treatment cells in the presence of increasing concentrations of daunorubicin. The $TI_{50}$ is seen here to be approximately 16 $\mu$g/l.

the ability of the cells to incorporate $^3$H-thymidine was measured. $^3$H-thymidine was added to the culture to give a final concentration of 0·5 $\mu$Ci/ml and incubation continued for a further 16 h. Cells were then harvested and $^3$H-thymidine uptake was measured according to Balkwill *et al.* (1974). The results of Balkwill *et al.* (1974) show that AML cells cultured under these conditions are replicating during the time we do our assay. This strongly suggests that the reduction we measure in $^3$H-thymidine incorporation reflects cell-killing, although we have not directly measured this. A typical incorporation curve is shown in the Figure. The drug concentration at which the incorporated counts were reduced by half (the $TI_{50}$) was taken as a guide to the *in vitro* drug sensitivity. The same approximate value was repeatedly obtained for all cells from the same patient, whether or not fresh or frozen cells were used. Tables I and II show the results obtained for our series of patients. Table I gives the results with pre-treatment cells; Table II shows the results with post-relapse cells. It can be seen that the *in vitro* $TI_{50}$ for most patients' cells lies between 10 and 30 $\mu$g/l. Only in the case of P.J. is the figure greatly different. In this case the cells are about 10 times more resistant to daunorubicin than any other cells tested by us. The post-relapse cells of A.H. and G.S. were just as sensitive as their pre-treatment cells, even though these patients, like P.J., had become clinically resistant to daunorubicin and cytosine arabinoside. We also find that there is no correlation between *in vitro* $TI_{50}$ and either the rate at which blasts

TABLE I.—In Vitro *and Initial Clinical Response to Daunorubicin (Pre-treatment Cells)*

| Patient | Initial white cell count × 10³/mm³ blood (% blasts) | White cell count 10 days after starting treatment × 10³/mm³ blood (% blasts) | $TI_{50}$ μg/l | Clinical outcome‡ |
|---|---|---|---|---|
| M.Bl. | 38 (75) | 1·0 (50) | 27 | C |
| J.M. | 29 (30) | 1·4 (0) | 16 | C |
| A.E. | 180 (90) | 11·0 (40) | 25 | C |
| M.C. | 225 (95) | 0·3 (36)* | 23 | N |
| M.W. | 30 (60) | 18·0 (40) | 15 | N |
| W.P. | 154 (96) | 14·0 (90) | 19 | C |
| A.H. | 69 (91) | 5·5 (62) | 13 | C |
| P.J. | 60 (95) | 1·0 (50) | 25 | C |
| T.L. | 28 (39) | 1·2 (12) | 16 | C |
| G.S. | 6·3 (41) | 5·0 (12) | 16 | N |
| S.J. | 33 (59) | 1·9 (30) | 18 | C |
| G.H. | 96 (74) | 1·9 (56)† | 20 | N |
| J.H. | 11 (90) | 1·0 (70) | 6 | N |
| R.M.C. | Daunorubicin not given clinically | | 13 | |
| E.E. | | | 20 | |
| E.W. | | | 25 | |
| E.O. | | | 23 | |
| M.B. | | | 21 | |
| S.G. | | | 23 | |

* Patient died on the 6th day after treatment began. The figures for the 6th day are given.
† Patient died on the 7th day after treatment began. The figures for the 7th day are given.
‡ C: complete remission; N: no complete remission.
The amount of daunorubicin received by a patient on the first day of treatment was calculated on the basis of his surface area. As a guide, it can be said that approximately 80 mg was received by a patient in any single dose.

TABLE II.—In Vitro *and Clinical Response to Daunorubicin of Patients after Relapse*

| Patient | Pre-treatment $TI_{50}$ μg/l | Post-relapse $TI_{50}$ μg/l* | Post-relapse white cell count × 10³/mm³ blood (% blasts) | Post-relapse white cell count after ten days treatment × 10³/mm³ blood (% blasts) | Clinical result |
|---|---|---|---|---|---|
| P.J. | 25 | 300, 300, 1000, 1000, 200, 200 | 240 (99) | 120 (99) | Resistant |
| A.H. | 13 | 38, 15, 15, 25 | 20 (99) | 40 (99) | Resistant |
| G.S. | 16 | 31, 25, 21 | 10 (99) | 10 (99) | Resistant |
| T.L. | 16 | 9, 23 | Not given daunorubicin again | | |
| S.J. | 18 | 20, 18 | Not given daunorubicin again | | |
| J.D. | — | 12, 21, 23 | Not given daunorubicin again | | |
| M.B. | 21 | 18 | Not given daunorubicin | | |
| A.W. | — | 13 | Not given daunorubicin | | |
| R.W. | — | 16 | Not given daunorubicin | | |
| I.M. | — | 23, 24, 18 | 94 (75) | 31 (4) | Complete remission |

* Separate estimations on samples taken from patient on different occasions.

are removed from the peripheral blood or the achievement of a complete remission.

Thus it is apparent that relative sensitivity in this *in vitro* test is not a reliable guide to the clinical susceptibility of AML patients to daunorubicin.

In the one case (P.J.) where clinical resistance paralleled the acquisition of *in vitro* resistance we examined the resistant cells to see whether they were also more resistant to other drugs. Table III shows the results of these experiments. It can be seen that in all cases P.J.'s cells are more resistant than those of W.P. and suggests that there has been

TABLE III.—In Vitro *Drug Resistance in P.J. and W.P.*

| Drug | Pre-treatment cells of W.P. $TI_{50}$ µg/l | Post-relapse cell of P.J. $TI_{50}$ µg/l |
|---|---|---|
| Daunorubicin | 19 | 250 |
| Cytosine arabinoside | 2 | 10 |
| Adriamycin | 150 | 1500 |
| Puromycin | 25 | 125 |

The post-relapse cells of P.J. are compared here with the pre-treatment cells of W.P. as there were no more of P.J.'s pre-treatment cells to compare them with.

TABLE IV.—In Vitro *Drug Resistance in a Number of AML Patients to Daunorubicin and Daunorubicin-DNA*

| Patient | Cells | $TI_{50}$ µg/l daunorubicin | $TI_{50}$ µg/l daunorubicin-DNA |
|---|---|---|---|
| P.J. | Post-relapse | 250 | 145 |
| W.P. | Pre-treatment | 21 | 38 |
| G.H. | Pre-treatment | 18 | 16 |
| T.L. | Pre-treatment | 14 | 15 |
| S.G. | Post-relapse | 19 | 25 |

The $TI_{50}$ is calculated with respect to the daunorubicin added to the cell cultures whether or not it was conjugated to DNA. This experiment has been repeated with essentially the same result.

a simultaneous acquisition of multiple drug resistance by P.J.'s cells. This is particularly interesting since P.J. had never been exposed clinically to adriamycin or puromycin although she had received cytosine arabinoside. It could be that this is due to a membrane change which might be by-passed by fixing the daunorubicin to a large polymer such as DNA (Sokal *et al.*, 1973). We therefore compared the *in vitro* sensitivity of P.J.'s cells and the cells of several other AML patients to daunorubicin conjugated to DNA and unconjugated daunorubicin. Table IV shows the results. It can be seen that to some extent conjugation of daunorubicin to DNA improves the ability of daunorubicin to reduce $^3$H-thymidine incorporation in P.J.'s cells *in vitro*. In no other patient's cells is this so.

### DISCUSSION

Our results suggest that the measurement of *in vitro* drug resistance in this system is not a reliable indication of the clinical response a patient will show to drug therapy. In this study, however, the number of patients tested is small.

In the single instance where drug resistance *in vitro* parallels the clinical picture we are struck by the simultaneous acquisition of resistance to several drugs. A similar cross-resistance phenomenon has previously been reported in tissue culture cells exposed to increasing amounts of drug *in vitro* (Minor, 1974). A possible explanation for this cross-resistance is that a membrane change has occurred affecting the transport of these drugs into the cells, and indeed membrane changes affecting daunorubicin transport have been reported. Ehrlich ascites cells exposed *in vitro* to daunorubicin undergo selection to produce a cell-line 5 times more resistant to daunorubicin than the parent cell-line (Danø, Frederiksen and Hellung-Larsen, 1972). This resistant cell has been shown to be resistant because of a permeability change and it appears that the drug-resistance in this instance is due to active expulsion of daunorubicin from the cells (Danø 1973). Attempts to demonstrate this effect in P.J.'s cells with $^3$H-puromycin have failed, however, owing to the fragility of the cells after culture.

Our evidence that P.J.'s cells are more susceptible *in vitro* to daunorubicin-DNA than to unconjugated daunorubicin is consistent with the acquisition of an altered permeability. This finding may indicate that in cases of clinical resistance paralleled by *in vitro* resistance the therapy might, with advantage, include drugs conjugated to DNA or some other large carrier molecule.

We have no experimental evidence concerning the mechanisms by which such variants arise. Unfortunately, although the life of AML cells cultured under these conditions can be as long as 2–3 months (Balkwill and Oliver,

1976) the ability of P.J.'s cells to be trypsinized and continue to replicate has proved inadequate to allow us to select and investigate revertants.

We wish to record our deep sorrow at the death of Professor Gordon Hamilton Fairley without whose support and encouragement this work would not have been possible.

We would also like to thank Dr J. M. A. Whitehouse and Dr R. T. D. Oliver for their invaluable help and advice.

### REFERENCES

BALKWILL, F., PINDAR, A. & CROWTHER, D. (1974) Factors Influencing Microculture of Leukaemia Cells. *Nature, Lond.*, **251**, 741.

BALKWILL, F. R. & OLIVER, R. T. D. (1976) Diagnostic and Prognostic Significance of Peripheral Blood Cultural Characteristics in Adult Acute Leukaemia. *Br. J. Cancer*, **33**, 400.

BEARD, M. E. J. & FAIRLEY, G. H. (1974) Acute Leukaemia in Adults. *Seminars in Haematology*, **11**, 5.

BUCKNER, D., GRAW, R. G., EISEL, R. J., HENDERSON, E. S. & PERRY, S. (1969) Leukapheresis by Continuous Flow Centrifugation (CFC) in Patients with Chronic Myelocytic Leukaemia (CML). *Blood*, **33**, 353.

CROWTHER, D., POWLES, R. L., BATEMAN, C. J. T., BEARD, M. E. J., GAUCI, C. L. WRIGLEY, P. F. M., MALPAS, J. S., FAIRLEY, G. H. & SCOTT, R. B. (1973) Management of Adult Acute Myelogenous Leukaemia. *Br. med. J.*, i, 131.

DANØ, K., FREDERIKSEN, S. & HELLUNG-LARSEN, P. (1972) Inhibition of DNA and RNA Synthesis by Daunorubicin in Sensitive and Resistant Ehrlich Ascites Tumour Cell *in Vitro*. *Cancer Res.*, **32**, 1307.

DANØ, K. (1973) Active Outward Transport of Daunomycin in Resistant Ehrlich Ascites Tumor Cells. *Biochim. biophys. Acta*, **323**, 466.

MINOR, D. P. (1974) A Study of Colchicine Resistance in Mammalian Cell Lines. Ph.D. Thesis, London University.

POWLES, R. L., BALCHIN, L. A., SMITH, C. & GRANT, C. K. (1973) Some Properties of Cryopreserved Acute Leukaemia Cells. *Cryobiology*, **10**, 282.

SOKAL, G., TROUET, A., MICHAUX, J. L. & CORNU, G. (1973) DNA-daunorubicin Complex: Preliminary Trials in Human Leukaemia. *Eur. J. Cancer*, **9**, 391.